# Push-Out Bond Strength of Endodontic Posts Cemented to Extracted Teeth: An In-Vitro Evaluation

**DOI:** 10.3390/ma15196792

**Published:** 2022-09-30

**Authors:** Syed Rashid Habib, Abdul Sadekh Ansari, Aleshba Saba Khan, Nawaf M. Alamro, Meshari A. Alzaaqi, Yazeed A. Alkhunefer, Abdulaziz A. AlHelal, Talal M. Alnassar, Abdulaziz S. Alqahtani

**Affiliations:** 1 Department of Prosthetic Dental Sciences, College of Dentistry, King Saud University, Riyadh 11545, Saudi Arabia; 2 Dentistry Hospital, King Saud University Medical City, Riyadh 11545, Saudi Arabia; 3 Department of Prosthodontics, Shahida Islam Dental College, Lodhran 59320, Pakistan; 4 College of Dentistry, King Saud University, Riyadh 11545, Saudi Arabia

**Keywords:** endodontic posts, dowel, fiber posts, prefabricated posts, titanium posts

## Abstract

(1) Background: An ideal bond strength between endodontic posts and root canal dentin is essential for optimal retention and good prognosis. This study aimed to evaluate the push-out bond strength (PBS) of prefabricated fiber and metal posts, luted with resin cement to natural dentin. (2) Methods: Extracted premolars with similar root dimensions were assigned into two groups of 30 each for the metal and fiber posts. Teeth were mounted in acrylic blocks exposing 2 mm of the coronal root. Teeth were subjected to endodontic treatment and post-space preparations. Two groups were further subdivided into three sub-groups (n = 10) according to the size of the posts (# 4, 5 and 6). Posts were cemented with resin cement. Specimens were sectioned into 4 mm slices and subjected to the PBS test. (3) Results: The mean PBS was similar for the metal and fiber posts bonded with resin cement, showing a statistically significant result. An increase in post size increased the bond strength initially, but a further increase in size did not show any marked difference. A total of 71.66% of tested specimens failed with the adhesive failure mode. (4) Conclusions: Metal posts showed slightly higher retention compared to the fiber posts, although the *p*-value was similar for both types. An increase in the size of posts showed increased retention. The most common mode of failure was adhesive failure between cement and dentin.

## 1. Introduction

The preservation of sound tooth structure will ensure the strength of the teeth and is critical for the long-term success of endodontically treated teeth [1]. Root canal treatment, followed by the preparation of the post space, results in a reduction in the remaining tooth structure, which compromises the strength of the teeth [2]. Factors such as the amount and quality of tooth structure, tooth position in the arch, anatomy, function, absence of a periapical infection, a well-condensed root filling, the root filling extending to 2 mm within the radiographic apex and not beyond, a satisfactory coronal restoration and use of a rubber dam during treatment may also contribute to the success of endodontically treated teeth [3]. 

Root canal treatment, when carried out on badly damaged teeth, makes them feebler and less resistant to cracks or fractures under occlusal loads or stresses [4,5]. After endodontic treatment, already weak and broken-down teeth need a reconstruction of walls and tooth structure before the provision of fixed dental prosthesis. The greater the damage to the walls of teeth, the more the chances of incorporating endodontic posts are considered to retain the build-up of the tooth before crowning [4,5,6,7]. Different post systems, i.e., metal, zirconia, fiber and composite, are commercially available [8,9]. The difference in rigidity of different post materials and the structure of the tooth may compromise the endurance limit of the tooth treated with an endodontic post [4,10]. With advancements in mechanical properties, along with the benefit of esthetic properties, fiber posts are being increasingly considered, as their rigidity is comparable to the dentin [5,10]. In addition to good mechanical properties, the prefabricated metal and fiber posts also possess excellent biocompatibility properties [11].

The most common type of failures seen in the teeth restored with endodontic posts is the loss of bond between the post and the tooth structure, retention loss, post fracture or fracture of the tooth [12,13]. Fiber posts have the benefit of preventing the tooth from fracturing by taking the impact, and the post may get fractured by itself [12]. The most repeated issue seen when the root-canal-treated tooth receives a fiber post is debonding [5,8,10].

Resin-based adhesive cements are more extensively being adopted for the cementation of different fixed dental prosthesis and endodontic posts [5]. Multiple adhesive systems, such as self-cure or dual-cure treatments, are available [7]. Few studies have reported that a self-adhesive resin-based system provides a simple technique for adhesion, thus limiting the possible drawbacks. Meanwhile, some studies have reported that the conventional or dual-cure resin system shows less solubility and better bonding with dentin [12].

The choice of an appropriate cement for luting the posts to dentin in the root canal is a challenge [4,8]. The strength of bond formed is affected by several factors; nevertheless, higher success rates have been reported with resin-based cements and fiber posts [5]. Many factors affect the bond strength of the posts by influencing the bond formed between cement–tooth and cement–fiber posts, as well as factors that lead to difficulties in bonding [4,5,6,7,8]. The factors to keep in consideration are the anatomy and preparation of the canal, dentin dehydration, choice of cement for luting, type of sealer used during endodontic treatment, direction of tubules of dentin in the canal at various levels, controlling contamination with saliva, working in indirect vision inside the canals, stress caused by polymerization contraction of the cement, density of layer of the cement and cyclic loads during mastication [4,5,14].

Over the years, few tests such as the micro-tensile strength evaluation, as well as the pull-out and push-out tests have been devised to assess the bond strength among the posts and root canal dentin [5,13]. Some drawbacks associated with micro-tensile and pull-out test, as well as more reliable results with push-out tests, make this method more widely acceptable to evaluate adhesive strength [7,12]. 

The push-out strength test, or PBS, was first advocated by Roydhouse11 in 1970, and it is being frequently used to evaluate the shear bond strength of resin-based cements to root dentin in relatively thick cross-sectional specimens of root-canal-treated teeth. In this method, a compressive load is applied to the sliced-root specimen in an apico-coronal direction to push the post coronally. The stress applied in this analysis is more uniform, and it gives a better estimation of bond strength as it imitates the clinical conditions by debonding in small sections of the root [15,16].

This study aimed to assess the PBS of fiber and metal posts luted with dual-cure resin-based cement to the natural dentin. The study also evaluated the PBS for different sizes of fiber and metal posts. In addition, analysis for the methods of failure among the cemented posts, cement and dentin was performed. The null hypothesis tested stated that there is no variation between the PBS of fiber versus metal posts, and there is no effect of post size on the PBS.

## 2. Materials and Methods

**Ethical Approval:**

As this research study was performed on extracted human teeth, an ethical approval was obtained from the institutional review board at King Saud University, Riyadh (Registration # E-21-5804) before commencement of the study. 

**Specimen Preparation:**

**Figure 1:**
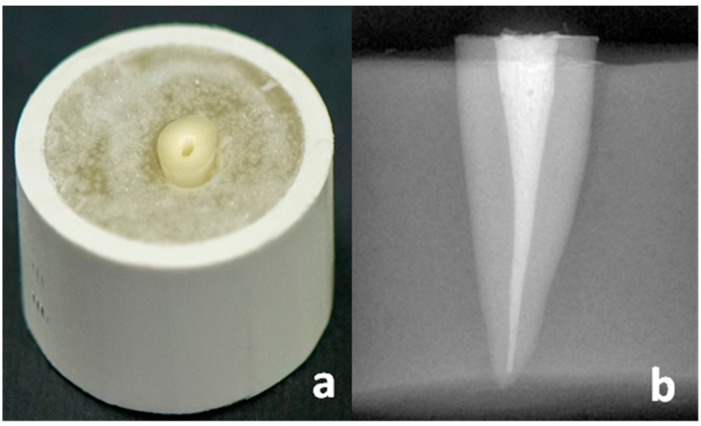
(**a**): Mounted tooth specimen sectioned at cemento-enamel junction; (**b**): Radiograph of specimen after endodontic treatment and before post space preparation.

Sixty mandibular first premolar teeth with caries-free, single-straight roots, as well as almost-similar root dimensions extracted for orthodontic purposes, were collected for the present study. Obtained teeth were cleaned with water and disinfected with 5.25% NaOCl solution and stored in distilled water at 37 °C for 24 h. NaOCl, which is easily available in dental setups, was used for disinfection as it is considered one of the most potent agents against a wide range of pathogens such as Gram-positive and Gram-negative bacteria, fungi, viruses and spores, and it is even reported to be effective against HIV present in the oral micro-biofilm [17]. Then, each tooth was mounted in a clear acrylic resin cylinder (Orthoplast, Vertex dental, AV Soesterberg, The Netherlands) of 2 × 2 cm dimensions, exposing the anatomic crown and 2 mm of the coronal portion of the root. To obtain the study specimens, anatomic crowns of all the teeth were removed by transversely sectioning the cemento-enamel junction with a diamond disc (NTI Sintered, Kerr Corporation, Brea, CA, USA) in a high-speed air-turbine hand piece (NSK, Nakanishi Inc., Shinohinata, Kanuma Tochigi, Japan) under copious water irrigation (Figure 1a). All the obtained specimen roots were subjected to endodontic treatment using Protaper Ni-Ti rotary instruments (size S1, S2, F1, F2; Dentsply Maillefer, Tulsa, OK, USA) in a high-torque endodontic motor (X-Smart, Dentsply Maillefer, Tulsa, OK, USA) up to ISO size 35 and 0.06 taper, following the crown-down technique. A total of 17% EDTA (Pulpdent, Watertown, MA, USA) and 5.25% sodium hypochlorite (Ogna, Milan, Italy) were used for irrigation. All the root canals were obturated with gutta-percha (Kerr Corporation, Brea, CA, USA) and an endodontic sealer (AH plus, Dentsply Maillefer, Tulsa, OK, USA), using the warm vertical condensation technique (System B, SybronEndo, Orange, CA, USA), and backfilled with thermo-plasticized gutta-percha using Obtura II (Spartan, Fenton, MO, USA) (Figure 1b). Then, specimens were stored in 100% relative humidity at room temperature for 24 h. Specimens were randomly divided into two groups of thirty each (Group-A = Metal post and Group-B = Fiber post), according to the type of post. Further, each group was subdivided into three subgroups of ten specimens each (n = 10/post sizes #4, #5, #6) according to the size of post used, as shown in Table 1.

**Figure 2:**
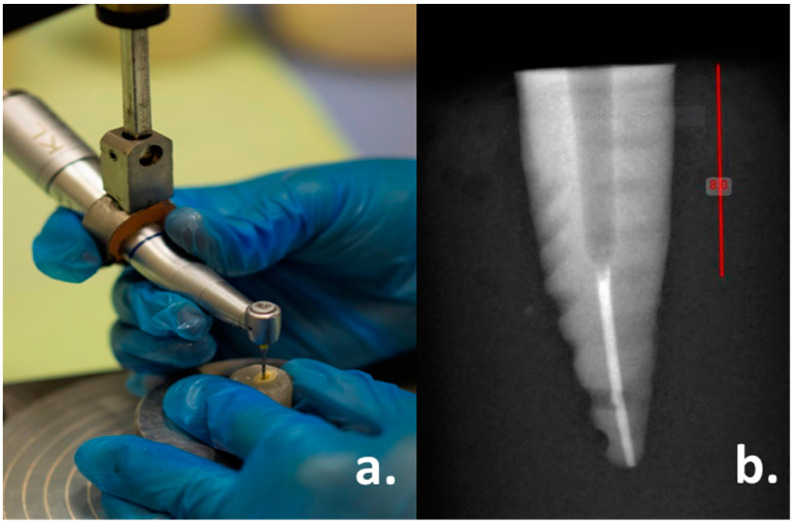
(**a**): Customized surveyor used during post space preparations; (**b**): Radiograph after post space preparation.

After 24 h of storage, all the filled root canals were prepared for post spaces using Peeso reamers (Pulpdent Corp, Watertown, MA, USA) of sizes #2, #3 and #4, sequentially, for each specimen to remove gutta-percha, and then the specimens in each group were prepared for the final post spaces using Parapost drills (Coltene/Whaledent Inc. Feldwiesenstrasse, Altstätten, Switzerland) of sizes #4, #5 and #6, respectively, as recommended by the manufacturer for the similar size of posts used. All the post spaces were prepared uniformly at a standardized depth of 8 mm, leaving 5 mm of apical gutta-percha intact under copious water irrigation with a slow-speed dental handpiece (NSK, Nakanishi Inc., Shinohinata, Japan) attached to a customized dental surveyor (J. M. Ney Co., Hartford, Connecticut, CT, USA) to guide the post space preparation, parallel to the long axis of the teeth. Periapical radiographs were taken for all the specimens before and after post space preparations to confirm the optimal post space preparations and to ensure no traces of gutta-percha and sealant remained in the post spaces (Figure 2a,b).

**Figure 3:**
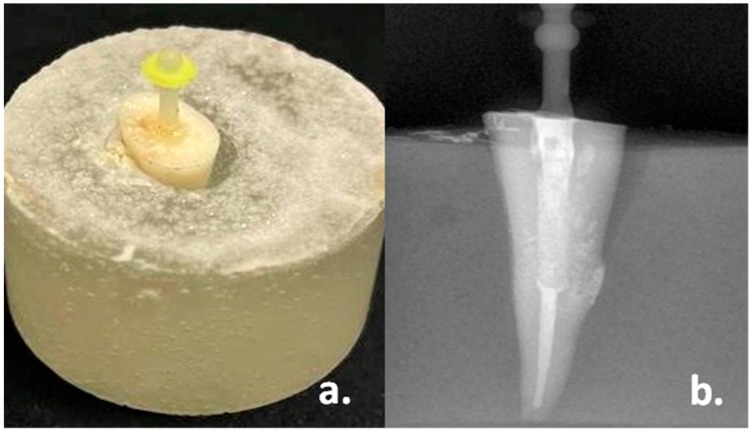
(**a**): Photograph of the specimen after cementation of post; (**b**): Radiograph of the specimen after cementation of post.

The cementation of endodontic posts was performed as per the recommendations of the manufacturer for each group. Root dentin in the prepared post spaces of all the specimens were etched with 37% orthophosphoric acid etching gel (DentoEtch, Itena-Clinical, Villepinte, France) for 5 s, cleaned with normal saline and dried using absorbent paper points. The posts were cleaned with 92.8% ethanol and silanized with Monobond N Primer (Ivoclar Vivadent AG, Schaan, Liechtenstein) before cementation. The prepared root canals were coated with Multilink primer (Ivoclar Vivadent AG, Schaan, Liechtenstein) using a microbrush, and any excess was blotted with absorbent paper points. Then, each post was cemented into the respective specimens using Multilink N (Ivoclar Vivadent AG, Schaan, Liechtenstein) dual-cure resin cement, as per the manufacturer’s instructions. The required amount of resin cement was dispensed into a post space through the intracanal tip attached to an automix syringe. Then, each post was inserted into the prepared post space with light finger pressure and a dental surveyor (J. M. Ney Company, Hartford, Connecticut, CT, USA) was used to guide the cementation of posts parallel to the long axis of the root. Extruded excess cement was removed with a microbrush, leaving the cement flush with the coronal portion of the root, and it was then polymerized with a visible light curing unit (XL 2500; 3M ESPE, St. Paul, MN, USA) for 40 s at 300 mW/cm^2^ intensity. Periapical radiographs were recorded to verify the post fit after cementation (Figure 3a,b). Then, all the specimens were stored in a distilled-water container at 37 °C for one week. 

**Slicing of specimens for Push-out test:**

**Figure 4:**
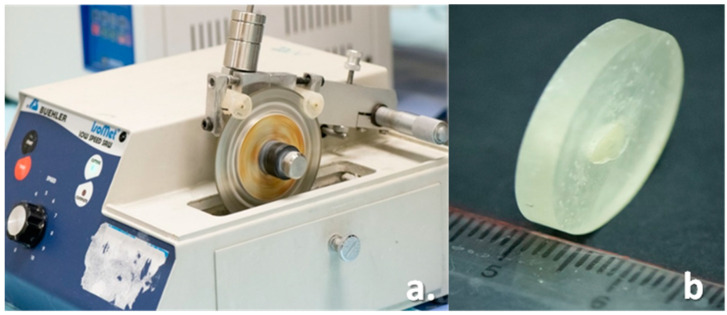
(**a**): Mounted specimen on Isomet precision machine for sectioning; (**b**): 4 mm slice of specimens obtained for push-out test.

The specimens were transversely sectioned into 4 mm thick slices using a precision cutting saw (Isomet Low Speed Saw, Buehler Ltd., Lake Bluff, IL, USA) at a speed of 150 rpm with a diamond cutting blade under copious water cooling. A first transverse cut was made in the coronal part of the root, measuring 4 mm from the cemento-enamel junction, and the second transverse cut was carried out at 4 mm apical to the first cut, discarding 5 mm of the apical part of the root. Two slices of 4 ± 0.1 mm thickness were obtained from each specimen corresponding to the coronal and middle portion of each root. A slice thickness of 4 mm was chosen to keep sufficient dentin thickness that can withstand the push-out load without fracturing the specimen (Figure 4a,b).

**Push-out test:**

**Figure 5:**
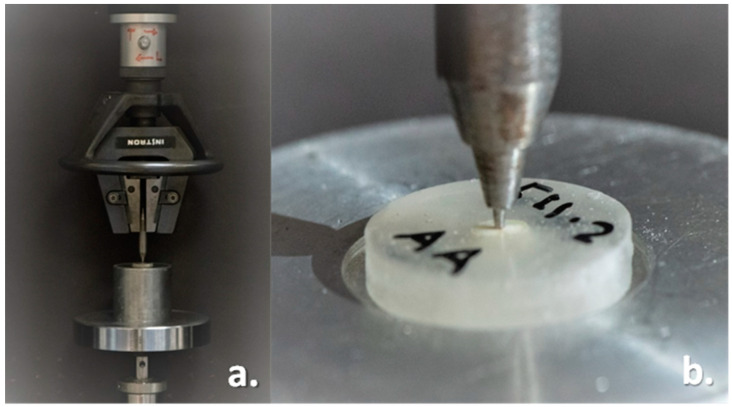
(**a**): Specimen attached to the universal testing machine for push-out test; (**b**): Close up view of the slice attached to push-out jig with plunger centered on the post.

Each 4 mm thick slice was subjected to a push-out bond strength test using a universal testing machine (Instron, model 8500 Plus; Dynamic Testing System; Instron Corporation.; Norwood, MA, USA.) at a cell load of 50 kg and a crosshead speed of 0.5 mm/min until the post was debonded (Figure 5a). Each slice was secured on a push-out jig, and the load was applied from the apico-coronal direction so as to push the post towards the broader part of the root, thus avoiding any limitations to the post movement due to the root canal taper. The metallic end of the push-out plunger, measuring 0.8 mm in diameter, was positioned vertically on the slice so that it was centered on the post without creating any stress in the surrounding dentinal walls (Figure 5b). A constant vertical static load was applied until failure occurred. The maximum load recorded at the point of extrusion of the post from the slice was considered the point of bond failure. The push-out bond strength of each slice was recorded in megapascal (MPa). After the push-out test, specimens were individually stored in dry containers. 

**Failure mode analysis:**

**Figure 6:**
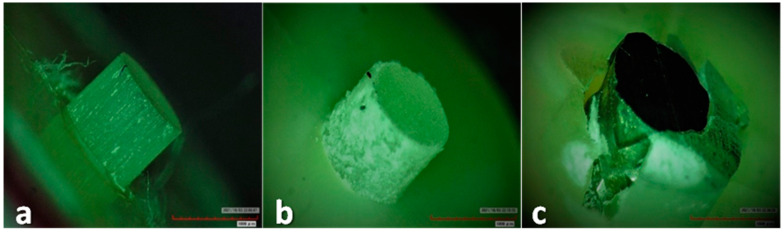
(**a**): Digital microscopic picture showing adhesive failure of cement–post interface; (**b**): Specimen showing adhesive failure of cement–dentin interfaces; (**c**): Specimen showing mixed failure.

After the push-out test, all the slices were evaluated under a digital microscope ((HIROX, KH-7700, Digital microscope system, Tokyo, Japan) at 50× magnification to analyze the failure modes. Each tested slice was observed under a digital microscope and failure modes were determined to be an adhesive failure within the cement–dentin interface, adhesive failure within the cement–post interface and mixed failure (Figure 6a,b). Based on the failure mode, the percentage of the failed specimens were calculated and recorded.

**Statistical analysis:**

All the collected data were tabulated and analyzed using SPSS (Version 23.0, SPSS, Chicago, IL, USA) analysis software. ANOVA and post-hoc Tukey tests were used for the statistical analysis of the collected data.

## 3. Results

A total sample size of N = 60 teeth was included in the study. Metal and fiber posts of three different sizes were bonded with dual-cure resin-luting cement. A total of 30 (50%) teeth received fiber posts and 30 (50%) received metal posts. Three sizes (# 4, 5 and 6) were used for each endodontic post system (Table 2). Size 5 posts showed the highest push-out bond strength for both types, i.e., metal and fiber. The mean push-out bond strength for both types of post was almost similar: 225.45 ± 132.94 for the metal posts and 225.29 ± 109.67 for the fiber posts. Slightly higher values were observed for the metal posts, and the results were statistically significant, depicting an increased bond strength with an increased post size as shown in Table 2.

The descriptive statistics show that, on comparison of push-out bond strength among different sizes of metal posts, a statistically significant increase in mean bond strength was observed when the size increased from 4 to 5. A slight rise in mean value was also seen in push-out bond strength when the size increased from size 4 to size 6. Mean push-out bond strength varied from 283.24 ± 119.65 for size 5 and 251.03 ± 158.19 for size 6, showing a slight further increase when considering the maximum values with standard deviation. Results were statistically significant (Table 2).

A comparison of push-out bond strength among variable sizes of posts in the fiber post system showed that significant results were observed when the size increased from size 4 to size 5, or from size 4 to 6, depicting an increase in surface area for bonding, thus increasing the retention of the post with increasing sizes. An increase in the size of fiber posts from size 5 to 6 did not show a marked increase in push-out bond strength of the fiber posts. Results, as analyzed by the ANOVA test, were statistically significant (Table 2 and Table 3).

On comparison of metal with fiber posts, the mean difference in POBS (push-out bond strength) was 19.61 for size 4 posts, −1.43 for size 5 posts and −9.69. for size 6 posts A group comparison of both posts systems showed a marked increase in mean values as size increased but the results were insignificant (Table 4). Both the systems, i.e., metal and fiber, showed similar patterns. Thus, an insignificant difference was noticed in the push-out bond strength of the metal versus fiber post systems when bonded with dual-cure resin-based luting cement.

### Analysis of Modes of Failure

**Figure 7:**
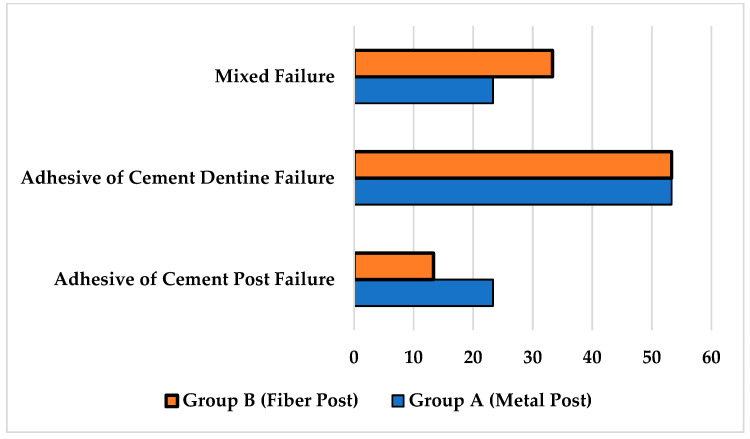
Graph showing analysis of failure modes for tested groups.

The analysis of methods of failure (Figure 7) revealed that more than half of the total failures were observed within the cement and dentin interface, with the adhesive failures of cement–dentin accounting for 53.33%. Meanwhile, 18.33% of the specimens had failures within the cement–post interface (adhesive failures of cement–post), and mixed failures accounted for 28.33%.

## 4. Discussion

In this research, the PBS of two different types of endodontic post systems and three variable sizes of each post system, bonded to natural teeth with resin-based cement, was evaluated and tested. In earlier published data, multiple methods, such as the pull-out and micro-tensile tests, have been performed to analyze the bond strength of endodontic posts cemented in the roots of natural teeth [5,18]. In the current study, the push-out test was applied to yield the gauging of the bond strength in two different parts of the root. This method put specimens under shear stresses between the dentin–cement and cement–post interfaces, which was comparable to clinical conditions [19,20]. In addition, a study by Goracci et al. [21] concluded that, when gauging the bond durability of fiber posts adhesively cemented to dentin of the root canal, the push-out test seemed to be more coherent and dependable than the micro-tensile technique [21]. Shear bond strength mainly depends on the degree and stability of the micromechanical intermesh and/or the chemical adherence between the dentin inside the root canal, the bonding cement and the post interface [21,22]. Therefore, for many reasons, the initial and long-term bond strengths achieved in clinical practice may be significantly less than optimal [21,22]. The adherence of an endodontic post cemented to the dentin in the root canal is largely dependent on various components, such as the type of luting cement used, the union among the cement–post and cement–dentin interface at different levels within the root canal, the physical or mechanical properties of the posts and the luting cements utilized and the impact of water-absorption on the cement, as well as the type, shape and surface characteristics of the post used [23,24]. Therefore, based on these observations, the investigation and correlation of the push-out bond durability of luted, prefabricated, parallel and serrated fiber or metal posts under homogenized conditions were performed in this research. A slight difference was seen in push-out bond strength where metal posts showed slightly better results, but the difference was statistically insignificant. This accepts the null hypothesis that there is no difference in the comparison between the bond strength of fiber posts versus the bond strength of metal posts.

In the current study, the variation in push-out bond strength amongst metal and fiber posts was insignificant, which is in accordance with other studies, such as those by Wang X and associates [22] and by R Sarkis et al. [25], which also reported similar results for metal and fiber posts [22]. R Sarkis et al. observed the performance of posts in clinical trials observed for five years [25,26]. Gbadebo and associates and Sterzenbach et al. also reported no variance in the performance of the two endodontic post systems, which is in accordance with the current study [27,28]. The basis for similarity may be that multiple other factors, i.e., frictional retention, shape of the post, micromechanical bond, etc. also play a role in the retention of posts, and not only the material for the post system is responsible for it [22]. 

The results of a study by Turker SA and associates reported that metal posts showed better bonding strength compared to fiber posts [22,29]. Other research by Singh A. et al. and Macros et al. reported that fiber endodontic posts had better bond durability in contrast to metal posts [22,30,31]. Schmitter et al. and King et al. reported a significant difference between the two post systems where fiber posts showed less bond strength and thus showed more debonding, and metal posts showed better bond strength and thus increases fractures in the metal post instead of debonding [22,32,33,34]. The findings of all these studies are in total contrast to the results of the current study. These differences in results might be due to the variation in the thickness of the cement layer as an intracanal site cannot be seen directly, thus resulting in a compromise in the adequate layer of thickness. This can lead to an increase in stress due to polymerization, as well as a reduced bond strength [35,36]. Although applying the cement in indirect vision with difficult access and a lack of adequate light to apply the cement makes the procedure technique sensitive, other factors such as the inherent nature of intra-canal dentin, the shape of the canal, the angulation and density of tubules in the dentin, smear layer, the remaining gutta-percha attached to walls of the canal and the role of the C-factor (proportion of bonded to un-bonded surface), result in variations in the results and standard deviation [19,29,30,31]. The choice of post diameter and taper according to shape of the canal also contributes to the variation in bond strength [17,21]. 

The current research showed that an increase in post size increased the push-out bond strength, as size was increased from 4 to 5 or 4 to 6 in both post systems. A study by Ulgey M et al. [37] showed that bond strength increased as the size of the post increased from 3 to 6, but the results were not statistically significant [36,37]. Another study by Amiri EM et al. [8] concluded the findings that the strength of the bond is higher in the cervical third in contrast to the apical region. This may be on the basis that, as surface area is increased for bonding, the bond strength is increased. Furthermore, a reduced number of tubules in the apical region compromised the bonding [8]. A thick layer of cement and an uncontrolled application of cement with different thicknesses in different canals also resulted in increased debonding [22,31]. A comparison of tapered posts versus cylindrical post- or pre-fabricated posts with custom posts also showed similar results in that that cylindrical posts and custom posts have a higher surface area available for bonding; thus, they yield better retention [26]. An increased surface roughness of posts or technique of application of cement in the canal might also affect the push-out bond durability of the posts by increasing the adherence [25,38].

Upon analysis of the mode of failure observed under a digital microscope, it was seen that 53.33% of failures were adhesive in nature and occurred between cement and dentin. Adhesive failure between cement and the post was 18.33% and 28.33%, respectively, which accounted for the mixed-type failure. Thus, adhesive failure was the most common failure seen in the present study. Similar results were reported by Aleisa K et al. [39] who reported adhesive failure to be 73.5% and thus predominant [39]. A study by Prisco et al. reported that debonding was mainly initiated by crack propagation at the junction of cement and post; thus, adhesive failure was considered to be the more common mode of failing [23]. The likely inference for this might be due to the remnants of gutta-percha, the smear layer or different thicknesses of cement in different areas [22]. The posts, i.e., fiber posts, have a modulus of elasticity near to dentin, which reduces the chances of tooth fracture; thus, failure is seen to be adhesive in nature.

Even though the adhesive failure is more likely to be observed under a digital microscope, other factors such as the type of cement, material of the post and its modulus, nature of prepared dentin in the canal and regions of stress concentration might lead to a variation in the mode of failure [39]. A study by Le Bell et al. observed that, as the bonding improved between posts and canals, the stress concentration shifted to the dentin instead of the junction, which might lead to a cohesive or mixed-failure types [40]. 

Despite an effort being made to standardize conditions to obtain accuracy in the results, a few factors cannot be controlled, such as the inherent nature of luting cement, polymerization stress, the role of the C-factor, the smear layer, technique sensitivity of cement application and the preparation of the post space, the remnants of sealer in the root canal and the nature of intra-canal dentin, which may have an impact on the outcome [22,39]. Another limitation was that this research was performed on extracted teeth, which might change characteristics of teeth due to ageing, thus affecting the bond strength [39]. Therefore, further research needs to be conducted in a clinical setting or by simulation of a clinical setting, i.e., by the artificial ageing of teeth, aside from using every possible mean to standardize the condition to obtain accurate responses from different post systems. Moreover, it was only performed on one type of tooth (premolars), so anterior as well as posterior teeth need to be incorporated in further research.

## 5. Conclusions

Fiber and metal endodontic posts bonded to extracted natural teeth with resin-based cement showed almost similar values of bond strength, with metal posts showing slightly higher values in contrast to that of fiber posts. An increase in the size of the post increased retention, which can be attributed to an increase in the bonding surface area. However, a further increase in post size limited the increase in strength of the bond at the expense of the root canal diameter or root dentin thickness. The most prevalent mode of failure observed was adhesive failure, i.e., failure among the dentin and cement, or between the post and cement.

## Figures and Tables

**Table 1:** Details of the tested post materials for each group in the present study.

Groups	Subgroup N-10	Post Material	Trade Name	Manufacturer	Post Size	Drill Used	Lot Number
Group A (Metal)	A1	Titanium post parallel serrated	Parapost XP	Coltene/Whaledent Inc. Feldwiesenstrasse, Altstätten, Switzerland	Size 4 Ø 0.9 mm Yellow P-784-4	Parapost Drill size 4	H17858
	A2	Titanium post parallel serrated	Parapost XP	Coltene/Whaledent Inc. Feldwiesenstrasse, Altstätten, Switzerland	Size 5 Ø 1.15 mm Red P-784-5	Parapost Drill size 5	H17858
	A3	Titanium post parallel serrated	Parapost XP	Coltene/Whaledent Inc. Feldwiesenstrasse, Altstätten, Switzerland	Size 6 Ø 1.4 mm Black P-784-6	Parapost Drill size 6	H17858
Group B (Fiber)	B1	Fiber post parallel serrated	Parapost fiber Lux Plus	Coltene/Whaledent Inc. Feldwiesenstrasse, Altstätten, Switzerland	Size 4 Ø0.9 mm Yellow PF1714	Parapost Drill size 4	H65570
	B2	Fiber post parallel serrated	Parapost fiber Lux Plus	Coltene/Whaledent Inc. Feldwiesenstrasse, Altstätten, Switzerland	Size 5 Ø 1.15 mm Red PF1715	Parapost Drill size 5	H65570
	B3	Fiber post parallel serrated	Parapost fiber Lux Plus	Coltene/Whaledent Inc. Feldwiesenstrasse, Altstätten, Switzerland	Size 6 Ø 1.4 mm Black PF1716	Parapost Drill size 6	H65570

**Table 2:** Descriptive statistics for the *PBS of the tested groups.

Post Type	Groups	N	Mean POBS	Std. Deviation	95% Confidence Interval for Mean		Minimum	Maximum	** Anova Results
					Lower Bound	Upper Bound			
Metal	Size 4	10	142.08	73.80	89.27	194.87	63.61	286.38	0.039
	Size 5	10	283.24	119.65	197.64	368.83	143.90	561.10	
	Size 6	10	251.03	158.19	137.86	364.20	77.21	600.67	
	Total	30	225.45	132.94	175.80	275.09	63.61	600.67	
Fiber	Size 4	10	130.46	38.10	103.20	157.71	76.29	189.72	0.001
	Size 5	10	284.67	120.78	198.26	371.07	126.88	463.08	
	Size 6	10	260.73	86.11	199.12	322.33	193.81	473.12	
	Total	30	225.29	109.67	184.33	266.24	76.29	473.12	

*PBS = push-out bond strength; ** *p* value was considered significant ≤ 0.05.

**Table 3:** Multiple comparisons and mean differences of the *PBS by Tukey HSD post-hoc tests.

Dependent Variable	Groups	Compared to	Mean Difference	Sig.	95% Confidence Interval	
					Lower Bound	Upper Bound
Metal	Size 4	Size 5	−141.16 **	0.040	−276.65	−5.67
		Size 6	−108.95	0.133	−244.44	26.52
	Size 5	Size 4	141.16 **	0.040	5.67	276.65
		Size 6	32.20	0.827	−103.28	167.69
	Size 6	Size 4	108.95	0.133	−26.52	244.44
		Size 5	−32.20	0.827	−167.69	103.28
Fiber	Size 4	Size 5	−154.20 **	0.002	−252.25	−56.16
		Size 6	−130.27 **	0.008	−228.31	−32.22
	Size 5	Size 4	154.20 **	0.002	56.16	252.25
		Size 6	23.93	0.818	−74.11	121.98
	Size 6	Size 4	130.27 **	0.008	32.22	228.31
		Size 5	−23.93	0.818	−121.98	74.11

*PBS = push-out bond strength; ** The mean difference was significant at *p* < 0.05.

**Table 4:** Group statistics (independent sample’s *t*-test) of the specimens based on the type of posts.

Post Size	Groups	N	Mean	Std. Deviation	Std. Error Mean	Mean Difference	Sig.	95% Confidence Interval of the Difference	
								Lower	Upper
Size 4	Metal	10	150.08	82.45	26.07	19.61	0.503	−40.73	79.95
	Fiber	10	130.46	38.10	12.04				
Size 5	Metal	10	283.24	119.65	37.83	−1.43	0.979	−114.32	111.52
	Fiber	10	284.67	120.78	38.19				
Size 6	Metal	10	251.03	158.19	50.02	−9.69	0.867	−129.36	109.96
	Fiber	10	260.73	86.11	27.23				

## Data Availability

Data are available on request from corresponding author.
